# Association between serum albumin levels and paroxysmal atrial fibrillation by gender in a Chinese population: a case–control study

**DOI:** 10.1186/s12872-022-02813-4

**Published:** 2022-08-28

**Authors:** Xia Zhong, Huachen Jiao, Dongsheng Zhao, Jing Teng

**Affiliations:** 1 The First Clinical Medical College, Shandong University of Traditional Chinese Medicine, Jinan, People’s Republic of China; 2 Department of Cardiology, Affiliated Hospital of Shandong University of Traditional Chinese Medicine, No. 42, Wenhua West Road, Lixia District, Jinan, Shandong People’s Republic of China

**Keywords:** Albumin, Paroxysmal atrial fibrillation, Gender, Blood lipid profiles

## Abstract

**Background:**

Hypoalbuminemia is linked to the emergence of cardiovascular events. However, there is an unclear association between serum albumin (ALB) and gender in paroxysmal AF patients. This retrospective study aimed to explore the association between ALB levels and paroxysmal AF by gender in a Chinese population.

**Methods:**

This study included patients with paroxysmal AF who were hospitalized consecutively in China from January 2019 to September 2021. Controls with sinus rhythm and without paroxysmal AF were matched (2:1) to cases by gender and age. Pearson correlation analysis was used to study the correlation between ALB and blood lipid profiles, multivariate regression models were performed to investigate the association between ALB and paroxysmal AF.

**Results:**

There were 305 patients with paroxysmal AF and 610 patients with controls included in this study. Low ALB in male with AF patients were significantly associated with paroxysmal AF (OR = 0.889, 95% CI 0.832–0.950). ALB was positively correlated with triglyceride (TG) (r = 0.212, p < 0.001), total cholesterol (TC) (r = 0.381, p = 0.002), low-density lipoprotein cholesterol (LDL-C) (r = 0.263, p < 0.001), and high-density lipoprotein cholesterol (HDL-C) (r = 0.329, p < 0.001).

**Conclusion:**

Low ALB in male patients is significantly associated with paroxysmal AF in a Chinese population. Monitoring for hypoalbuminemia in men might help reduce the incidence of paroxysmal AF.

## Introduction

Atrial fibrillation (AF) with a lifetime risk of about 25% represents an increasing public health concern, the most common clinical arrhythmia encountered in clinical practice, currently affecting almost 33 million subjects worldwide accompanied by growing morbidity and mortality especially in developed countries [1, 2]. Stroke and heart failure are serious possible consequences of AF [3], as well as an increased risk of embolism and death [4, 5]. Despite the introduction of therapeutic strategies such as antiarrhythmic drugs, electrical cardioversion, and catheter ablation, it seems that we are still far from highly effective treatment, with only a 66.6% success rate of a single operation for paroxysmal AF [3, 6, 7]. The era of AF prevention has come, and the management of risk factors has become a new paradigm of AF intervention [8]. Although recent guideline hasn’t recommended the use of specific biomarkers for AF [9], it has proved to be a valuable perspective for specific serum biomarkers to be used to understand and manage AF [10].

Serum albumin (ALB) has many biochemical properties [11], including anti-inflammatory, antioxidant, anticoagulant, antiplatelet aggregation, and colloid osmotic effect. Evidence is growing that hypoalbuminemia is considered a modifiable risk factor associated with cardiovascular events [12–14], mainly due to malnutrition and inflammation [15–17]. Evidence suggests that inflammation and oxidative stress are key to initiating and maintaining AF [18, 19]. Given the anti-inflammatory and antioxidant biochemical properties of serum albumin, we speculate that hypoalbuminemia could act as an important modifiable risk factor for AF [20, 21]. Recently, a prospective cohort study from 12,833 participants in the USA reported that serum ALB was independently inverse related to incident AF in a linear pattern [22]. Another earlier prospective cohort study from 8,870 participants in the Copenhagen City Heart Study (CCHS) also indicated that serum ALB was inversely associated with the risk of AF only in women (HR 0.47, 95% CI 0.28–0.77) [23]. Consequently, it could be speculated that hypoalbuminemia may be involved in the initiation and maintenance of AF; however, it is unclear if the results are the same in the paroxysmal AF population. Moreover, the gender relationship between serum ALB and AF has not been fully explored.

Hereby, in this case–control study, we aim to characterize the association between the ALB levels and paroxysmal AF by gender in 915 patients from China. We hypothesized that low serum ALB is associated with paroxysmal AF, the confirmation of this hypothesis has significant clinical value because serum ALB is adjustable.

## Methods

### Study population

This study included patients with paroxysmal AF who were hospitalized consecutively from a single center in China between January 2019 and September 2021 in the electronic medical record system. The inclusion criteria for cases were as follows: (1) patients from the community who were hospitalized for less than 21 days, lived a healthy lifestyle through medical history, and a normal nutritional status through the subjective global assessment (SGA) questionnaire. (2) patients aged 28–80 years. (3) Paroxysmal AF was diagnosed by clinicians and clearly documented in the electronic medical record. The exclusion criteria were as follows: (1) cardiac surgery, valvular disease, and heart failure. (2) peripheral vascular disease, prior thromboembolism, and major bleeding. (3) severe hepatic or renal dysfunction on record. (4) malignancy, hyperthyroidism, or gout. (5) pregnant woman. (6) use of uric acid-lowering drugs. Controls were also routine hospital inpatients, most of whom lived in the communities. Controls with sinus rhythm and without paroxysmal AF were matched (2:1) to cases by gender and age. In the current study design, we initially included a total of 2000 patients, including 354 patients with paroxysmal AF, 27 patients were excluded due to abnormal nutritional status based on SGA assessment, and 22 patients met other exclusion criteria. Finally, we included 305 patients with paroxysmal AF and 610 age- and sex-matched controls. This study was based on the principles of the Helsinki Declaration and approved by the Medical Research Ethics Committee of the Affiliated Hospital of Shandong University of Traditional Chinese Medicine. Because the data are anonymized, the Ethics Committee of Affiliated Hospital of Shandong University of Traditional Chinese Medicine (NO.20200512FA62) waived the need for informed consent.

### Study variables

Demographic and clinical variables included gender, age, AF comorbidities (coronary heart disease (CHD), hypertension, and diabetes), and laboratory variables (ALB, triglyceride (TG), total cholesterol (TC), low-density lipoprotein cholesterol (LDL-C), high-density lipoprotein cholesterol (HDL-C), apolipoprotein A1 (APOA1), serum apolipoprotein B (APOB), lipoprotein (a) [La(a)], serum creatinine (Scr), serum uric acid (SUA), aspartate aminotransferase (AST), and alanine aminotransferase (ALT). These data were obtained during routine hospital procedures and then reviewed by us through the hospital's electronic medical record system. All disease diagnoses were made by professional cardiologists and all serum indicators were determined by hospital laboratories.

### Definition and identification of paroxysmal AF

According to the reported guidelines [24], paroxysmal AF was defined as atrial fibrillation that was able to terminate spontaneously or by intervention within 7 days of onset. All patients with paroxysmal AF were identified and diagnosed by specialized clinicians.

### Classification of ALB levels and definition of hypoproteinemia

The serum ALB level conversion standard was as follows: 1 g/L = 0.1 g/dL. Levels of serum ALB were classified by tertiles. Serum ALB level in male patients was classified into 3 categories: < 37.9 mg/dL, 37.9–41.7 mg/dL, and > 41.7 mg/dL. Likewise, serum ALB level in female patients was classified into 3 categories: ALB level < 37.7 mg/dL, 37.7–41.1 mg/dL and > 41.1 mg/dL. As previously reported [25], ALB level < 3.5 g/dL was indicated as hypoproteinemia.

### Statistical analysis

All analyses were conducted in SPSS software (version 26.0, SPSS Inc., Chicago, IL, USA) and GraphPad Prism software (version 9.0.0). Continuous data were expressed as mean ± standard deviations (SD) or medians and interquartile ranges (IQR) where appropriate. Categorical data were presented as numbers and percentages. Baseline characteristics were compared between groups by Chi-squared tests for categorical variables and T-tests or Mann–Whitney U tests for continuous variables. Serum ALB was modeled as a continuous variable and was divided into tertiles based on sample distribution. The differences among the three groups of variables were analyzed by variance (ANOVA). Pearson correlation analyses were performed to analyze the correlation between ALB and blood lipid profiles. We used multivariable Binary Logistic Regression models to investigate the association between Serum ALB and AF. Model1 has not been adjusted. Model2 adjusted for CHD, hypertension, and diabetes. Model3 adjusted for TG, TC, LDL-C, HDL-C, APOA1, APOB, AST, SCr, and SUA. Model4 further adjusted for all these factors. A P value < 0.05 indicated significance, the tests were 2-sided.

## Results

### Study Population

Table 1 showed the clinical characteristics of the study population. This study included 305 patients with paroxysmal AF and 610 matched controls without paroxysmal AF. Compared with the control group, ALB levels in male and female patients with paroxysmal AF were significantly lower (p < 0.001). Patients with paroxysmal AF had significantly lower levels of TG, TC, LDL-C, HDL-C, APOA1, and APOB (P < 0.05), and significantly higher levels of AST, Scr, and SUA (P < 0.05). Additionally, the paroxysmal AF group had more patients with CHD, hypertension, diabetes, and hypoproteinemia than the controls (P < 0.05), regardless of gender.

**Table 1:** Baseline characteristics of paroxysmal AF group and controls

Variable	All (N = 915)			Men (N = 487)			Women (N = 428)		
	Paroxysmal AF group (N = 305)	Control group (N = 610)	P value	Paroxysmal AF group (N = 174)	Control group (N = 313)	P value	Paroxysmal AF group (N = 131)	Control group (N = 297)	P value
Age, years	66.24 ± 10.69	67.07 ± 11.46	0.291	64.75 ± 11.61	63.91 ± 12.23	0.460	68.24 ± 9.02	70.40 ± 9.53	0.015*
CHD, n (%)	253 (82.95)	144 (23.61)	< 0.001*	139 (79.89)	60 (19.17)	< 0.001*	114 (87.02)	84 (28.28)	< 0.001*
Hypertension, n (%)	175 (57.38)	206 (33.77)	< 0.001*	94 (54.02)	93 (29.71)	< 0.001*	81 (61.83)	113 (38.05)	< 0.001*
Diabetes, n (%)	94 (30.82)	99 (14.75)	< 0.001*	49 (28.16)	51 (16.29)	< 0.001*	45 (34.35)	48 (16.16)	< 0.001*
TG, mmol/L	1.01 [0.76–1.42]	1.13 [0.82–1.51]	0.023*	0.97 [0.71–1.35]	1.13 [0.83–1.64]	< 0.001*	1.10 [0.86–1.53]	1.12 [0.81–1.43]	0.425
TC, mmol/L	4.22 ± 1.17	5.00 ± 1.10	< 0.001*	3.98 ± 1.01	4.83 ± 1.06	< 0.001*	4.53 ± 1.29	5.19 ± 1.12	< 0.001*
LDL-C, mmol/L	2.52 ± 0.97	2.96 ± 0.87	< 0.001*	2.39 ± 0.79	2.96 ± 0.87	< 0.001*	2.70 ± 1.15	3.05 ± 0.90	0.002*
HDL-C, mmol/L	1.08 ± 0.35	1.19 ± 0.30	< 0.001*	1.02 ± 0.32	1.19 ± 0.30	< 0.001*	1.16 ± 0.37	1.29 ± 0.30	< 0.001*
APOA1, g/L	1.12 ± 0.28	1.22 ± 0.25	< 0.001*	1.05 ± 0.27	1.22 ± 0.25	< 0.001*	1.21 ± 0.28	1.30 ± 0.24	0.002*
APOB, g/L	0.83 ± 0.57	0.98 ± 0.25	< 0.001*	0.82 ± 0.71	0.98 ± 0.25	0.004*	0.85 ± 0.27	0.98 ± 0.26	< 0.001*
Lp (a), mg/L	15.50 [8.15–32.90]	14.40 [6.88–28.10]	0.123	14.50 [7.70–27.50]	12.10 [5.60–23.40]	0.021*	16.80 [8.40–36.80]	17.50 [8.40–33.30]	0.706
AST, U/L	20.00 [16.00–26.00]	18.00 [15.00–22.00]	0.002*	20.00 [17.00–27.00]	19.00 [16.00–24.00]	0.032*	19.00 [14.00–25.00]	18.00 [15.00–21.00]	0.083
ALT, U/L	17.00 [13.00–26.00]	17.00 [13.00–23.00]	0.316	18.00 [13.00–27.00]	19.00 [14.00–26.00]	0.912	15.00 [11.00–25.00]	15.00 [12.00–19.50]	0.503
SCr, μmoI/L	75.78 ± 54.76	64.70 ± 20.88	0.001*	76.00 [68.00–88.00]	71.00 [64.00–79.00]	< 0.001*	60.00 [52.00–72.00]	54.00 [48.00–61.00]	< 0.001*
SUA, mg/dL	5.56 ± 1.97	5.27 ± 1.49	0.024*	5.76 ± 1.96	5.27 ± 1.49	< 0.001*	5.28 ± 1.97	4.62 ± 1.26	0.001*
ALB, g/L	37.77 ± 4.86	40.22 ± 3.99	< 0.001*	37.43 ± 5.04	40.56 ± 4.08	< 0.001*	38.22 ± 4.58	39.87 ± 3.87	< 0.001*
Hypoproteinemia, n (%)	81 (26.56)	38 (10.17)	< 0.001*	52 (29.89)	26 (8.31)	< 0.001*	29 (22.14)	29 (9.76)	< 0.001*

Data were presented as mean ± SD or n (%)

*Statistically significant value (P < 0.05)

**Fig. 1:**
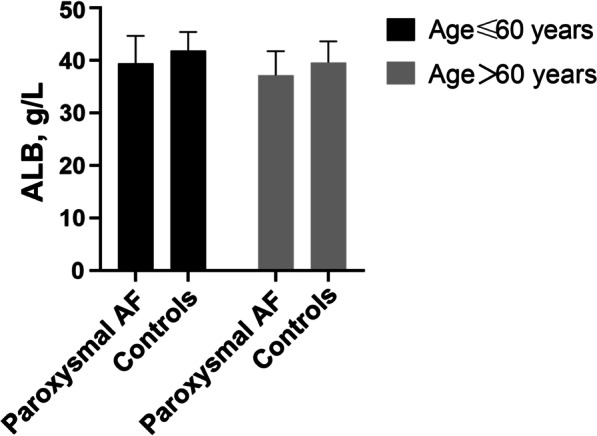
ALB levels in patients with paroxysmal AF and controls by age. Compared with controls, ALB levels in those aged ≤ 60 years (39.48 ± 5.20 vs.41.91 ± 3.54 g/L, P < 0.001) and aged > 60 years (37.19 ± 4.59 vs. 39.66 ± 3.98 g/L, P < 0.001) patients with paroxysmal AF were significantly lower

Figure 1 showed the differences in ALB levels by age in patients with paroxysmal AF and controls. Compared with controls, ALB levels in aged ≤ 60 years and aged > 60 years patients with paroxysmal AF were significantly lower (P < 0.001).

### Association between ALB and paroxysmal AF

Table 2 showed the association between ALB and paroxysmal AF. After adjustment for all confounding factors, ALB in male patients was significantly associated with paroxysmal AF (odds ratio [OR] 0.889, 95% confidence interval [CI] 0.832–0.950; p < 0.001).

**Table 2:** Association between ALB and paroxysmal AF

	Total		Men		Women	
	OR (95% CI)	P value	OR 95% CI	P value	OR 95% CI	P value
*Total*						
Model1	0.878 (0.849–0.908)	< 0.001*	0.857 (0.819–0.897)	< 0.001*	0.907 (0.862–0.955)	< 0.001*
Model2	0.906 (0.871–0.943)	< 0.001*	0.880 (0.834–0.928)	< 0.001*	0.944 (0.889–1.002)	0.060
Model3	0.893 (0.856–0.930)	< 0.001*	0.887 (0.839–0.937)	< 0.001*	0.912 (0.850–0.978)	0.009*
Model4	0.907 (0.864–0.953)	< 0.001*	0.889 (0.832–0.950)	< 0.001*	0.931 (0.858–1.011)	0.091

Model1: crude, no adjustment

Model2: adjusting for CHD, hypertension, and diabetes

Model3: adjusting for TG, TC, LDL-C, HDL-C, APOA1, APOB, AST, SCr, and SUA

Model4: adjusting for all these factors

*Statistically significant value (P < 0.05)

### The difference in ALB levels among the comorbidities of paroxysmal AF

Table 3 showed the difference in ALB levels among the comorbidities of paroxysmal AF. The results suggested that there was no significant difference in ALB levels among the comorbidities of paroxysmal AF (p > 0.05), regardless of gender.

**Table 3:** Complication difference in the association between ALB levels and paroxysmal AF

Variable	All			Men			Women		
	n	ALB, g/L	P value	n	ALB, g/L	P value	n	ALB, g/L	P value
paroxysmal AF + hypertension	175	38.07 ± 4.46	0.746	94	37.71 ± 4.68	0.764	81	38.48 ± 4.17	0.847
paroxysmal AF + CHD	253	37.75 ± 4.67		139	37.45 ± 4.70		114	38.12 ± 4.64	
paroxysmal AF + diabetes	94	37.71 ± 5.07		49	37.09 ± 5.41		45	38.39 ± 4.64	

Data were presented as mean ± SD

### Correlation between ALB and blood lipid profiles in patients with paroxysmal AF

**Fig. 2:**
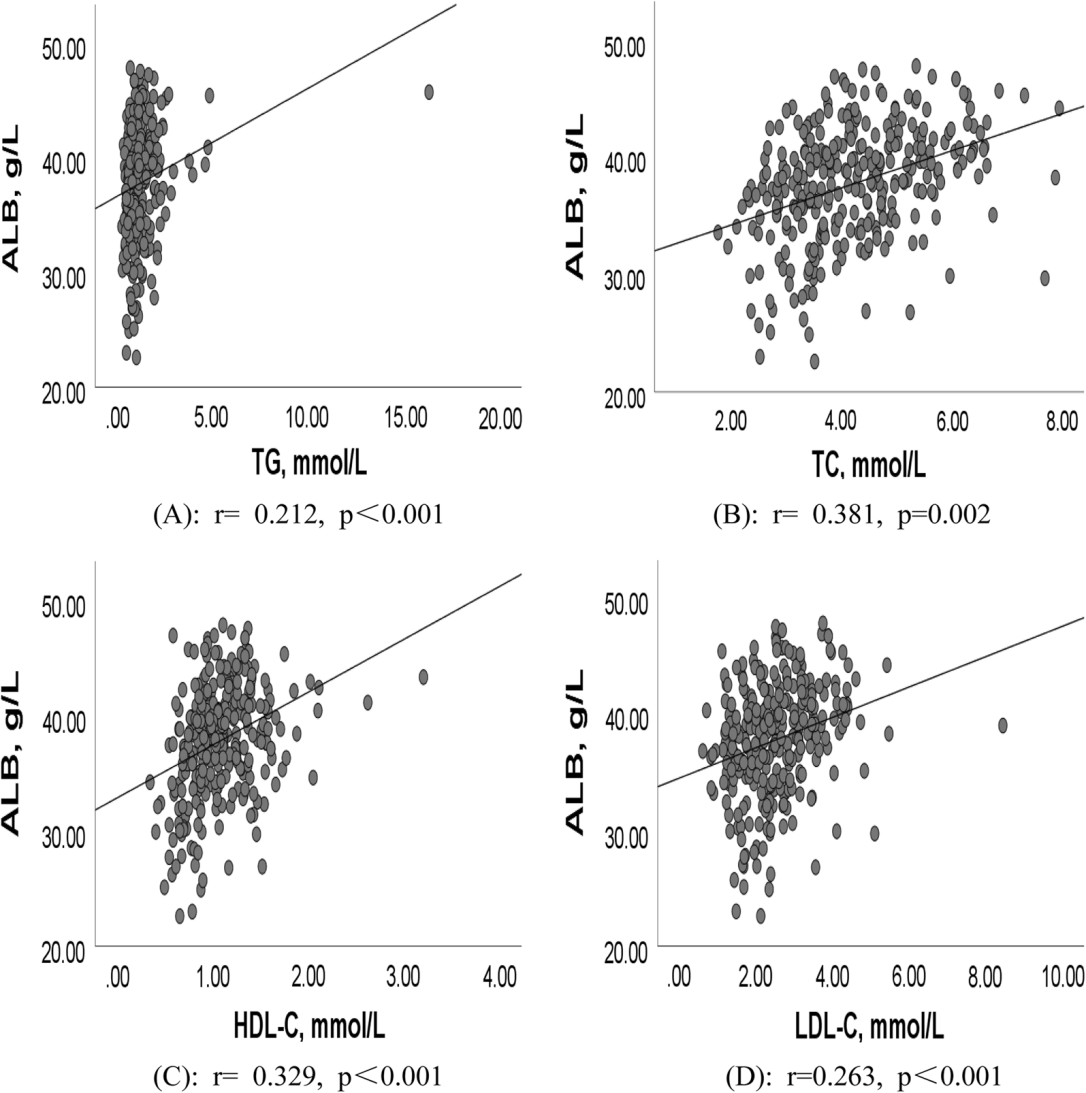
Correlation between ALB levels and blood lipid profiles in paroxysmal AF patients. **A** Correlation between ALB levels and TG in paroxysmal AF patients (r = 0.212, p < 0.001). **B** Correlation between ALB levels and TC in paroxysmal AF patients (r = 0.381, p = 0.002). **C** Correlation between ALB levels and HDL-C in paroxysmal AF patients (r = 0.329, p < 0.001). **D** Correlation between ALB levels and LDL-C in paroxysmal AF patients (r = 0.263, p < 0.001)

Figure 2 showed the correlation between ALB levels and blood lipid profiles in paroxysmal AF patients. The results showed that there was a positive correlation between ALB levels and blood lipid profiles in patients with paroxysmal AF, including TG (r = 0.212, p < 0.001), TC (r = 0.381, p = 0.002), HDL-C (r = 0.329, p < 0.001), and LDL-C (r = 0.263, p < 0.001).

### The association between ALB levels and blood lipid profiles in patients with paroxysmal AF by gender

Table 4 showed the subgroup analysis of the association between ALB levels and blood lipid profiles in paroxysmal AF patients. The results indicated that lower ALB in male and female patients with paroxysmal AF had lower TC and LDL-C (p < 0.001). Additionally, lower ALB in male patients with paroxysmal AF had lower TG (p < 0.05) and lower HDL-C in female patients (p < 0.05).

**Table 4:** The association between ALB levels and blood lipid profiles in patients with paroxysmal AF by gender

Variable	Men (n = 174)				Women (n = 131)			
	< 37.9 mg/dL	37.9–41.7 mg/dL	> 41.7 mg/dL	P value	< 37.7 mg/dL	37.7–41.1 mg/dL	> 41.1 mg/dL	P value
Number, n	86	54	34		56	40	35	
TG, mmol/L	0.98 ± 0.52	1.15 ± 0.51	1.38 ± 0.82	0.004*	1.04 ± 0.46	1.62 ± 1.02	1.74 ± 2.58	0.051
TC, mmol/L	3.64 ± 0.99	4.26 ± 0.86	4.41 ± 0.97	< 0.001*	3.70 ± 1.00	4.91 ± 1.34	5.11 ± 1.23	< 0.001*
LDL-C, mmol/L	2.16 ± 0.77	2.56 ± 0.72	2.68 ± 0.82	< 0.001*	2.27 ± 0.82	3.07 ± 1.46	2.99 ± 0.99	< 0.001*
HDL-C, mmol/L	1.08 ± 0.41	1.07 ± 0.33	1.09 ± 0.21	0.967	1.02 ± 0.31	1.16 ± 0.29	1.37 ± 0.44	< 0.001*

Data were presented as mean ± SD

*Statistically significant value (P < 0.05)

## Discussion

To our knowledge, this study is the first to evaluate the association between serum ALB and paroxysmal AF by gender in the Chinese population. Our current finding suggested that low serum low ALB levels in male patients were associated with paroxysmal AF; there was a positive correlation between ALB levels and blood lipid profiles in patients with paroxysmal AF. Nevertheless, further analyses didn’t support a significant difference in ALB levels among the comorbidities of paroxysmal AF. These findings imply that it would be interesting to monitor clinical hypoalbuminemia for clinicians to detect paroxysmal AF. We reported herein an association between low serum ALB levels and paroxysmal AF.

A recent dose–response meta-analysis reported that low serum ALB level was related to an increased risk of AF [26]. Liao et al. [22] conducted a large-scale epidemiological and mendelian randomization (MR) study to evaluate the causal influence of the serum ALB and incident AF; their results demonstrated that although serum ALB was inversely related to the incidence of AF after multiple adjustments, no evidence of a causal relationship between serum ALB levels and AF was found in Mendelian randomization (MR) analysis. Our current study, similar to this study, adjusted for several potential confounders such as CHD, hypertension, diabetes, SCr, SUA, and blood lipid profiles; moreover, the results of our study were supported by this study reporting that serum ALB was inversely associated with AF. Van et al. [27] performed a prospective cohort study to assess the relationship between serum ALB levels and new-onset AF (NOAF) in patients admitted to the ICU; similar to our study, they also analyzed data on hospitalized patients; their main result suggested that low serum ALB was related to the occurrence of NOAF; interestingly, they also found a relationship between low serum ALB land the number of onsets of NOAF; these results are also well in alignment with our main finding. Although these studies imply a close relationship between low serum ALB and AF, paroxysmal AF, which was often not adequately considered. A small retrospective study based on 32 consecutive patients and 32 age- and sex-matched paroxysmal supraventricular tachycardia patients from China reported that paroxysmal AF was associated with decreased serum ALB and hypoalbuminemia was an independent risk factor for paroxysmal AF (P = 0.0129, OR = 0.773) [28]. Compared with our study, the study design method of this study was similar, but our study had a larger sample size; we further analyzed the relationship between serum ALB and gender in patients with paroxysmal AF; meanwhile, we also adjusted for more confounders in the multivariable regression models and analyzed the correlation between serum ALB and blood lipids profiles in patients with paroxysmal AF.

The gender relationship between serum ALB and AF has not been fully explored. A prospective cohort design based on the Copenhagen City Heart Study reported that serum ALB levels only in females were inversely related to the risk of AF (hazard ratio: 0.47, 95% CI 0.28–0.77), which was inconsistent with our current results reporting that low serum low ALB levels in male patients were associated with paroxysmal AF. The main possible reason was the heterogeneity of the study population, it is clear that our study design relied on hospitalized patients in China, not the general population; more importantly, we also reliably differentiated paroxysmal AF from patients with AF; certainly, the influence of several potential unmeasured confounding factors can’t be ruled out. Admittedly, it would be interesting to perform a prospective cohort design to evaluate the association between serum ALB levels and paroxysmal AF in the future.

According to a previous review [29], hypoalbuminemia contributes to the pathological progression of cardiovascular events mainly through inflammation, oxidative stress, and platelet aggregation, as well as pulmonary and myocardial edema. Inflammation and oxidative stress have been considered to be two central mediators of atrial remodeling including electrical remodeling and structural remodeling for AF [30–32]. Evidence suggests a close relationship between low serum ALB and inflammatory markers. A previous study based on 4434 patients reported that serum ALB levels were negatively associated with CRP levels (r = -0.311) and white blood cell levels (r = -0.157) [33]. Acute and chronic inflammation affects serum ALB by altering liver protein metabolism and inducing capillary leakage [34–36]; monocyte products reduce serum ALB production during inflammation, which might be the underlying mechanisms [37]. Meanwhile, the association between hypoalbuminemia and oxidative stress has also been demonstrated [38]. Moreover, it has been shown that serum ALB has a unique biochemical structure, which forms an extracellular antioxidant defense [39]. Additionally, it must be mentioned that as an important marker of malnutrition, low ALB may also affect the proportional loss of myocardium and electrophysiological stability [28, 40, 41]. In the present study, although we didn’t evaluate the specific nutritional status indexes for hospitalized patients, we designed strict inclusion criteria, that is, patients from the community who were hospitalized for a short period of time had normal nutritional status. Therefore, we believe that it is essential to explore malnutrition-related AF in the future.

In the past, Annoura M et al. [40] reported the “cholesterol paradox”, suggesting that low TC and TG were associated with paroxysmal AF. Likewise, Psaty BM et al. [42, 43] also found the relationship between low blood lipid profiles and AF. Our current results suggesting low blood lipid profiles in paroxysmal AF are supported by this conclusion. Interestingly, we also observed that there was a positive correlation between ALB levels and blood lipid profiles in patients with paroxysmal AF. Even more so, it is possible that lower serum ALB and blood lipid profiles were jointly involved in the pathologic progression of paroxysmal AF. Although we were unable to provide more evidence for this hypothesis, it must be mentioned low HDL-C in our paroxysmal AF patients. Therefore, it could be speculated that the weaker anti-inflammatory and antioxidant properties of HDL-C can’t prevent the formation of AF matrix and some potential risk factors. Nevertheless, further subgroup analysis suggested the different associations between ALB levels and blood lipid profiles in paroxysmal AF by gender. Specifically, lower ALB in male and female patients with paroxysmal AF had lower TC and LDL-C; lower ALB in male patients with paroxysmal AF had lower TG and lower HDL-C in female patients. Gender differences in blood lipids and atrial electrophysiological characteristics [44, 45] might explain our findings.

It is well-known that hypertension, CHD, and diabetes are important risk factors for AF [46]. In our study, there was a higher incidence of hypertension, CHD, and diabetes because our hospitalized patients were from cardiology departments. We adjusted for these confounders as much as possible and analyzed the difference in ALB levels among these comorbidities of paroxysmal AF. Nevertheless, the current results didn’t support a significant difference in ALB levels among the comorbidities of paroxysmal AF. Clearly, this result wasn’t in disagreement with our findings. Certainly, it is necessary to further explore the specific relationship between serum ALB levels and isolated paroxysmal AF.

### Study limitation

Several potential study limitations warrant discussion. First, the small study population was recruited retrospectively from a single center in China, which was underrepresented and may have introduced selection bias. Therefore, further multicenter studies with large sample sizes are required. Second, the causal association between ALB levels and paroxysmal AF could not be determined due to the case–control design. Thus, further prospective cohort studies also are required. Third, low ALB, as an acute phase reactant, could be related to long-term inflammation [22]. Several inflammation markers, such as C-reactive protein (CRP) and interleukin-6 (IL-6), weren’t measured in our study at baseline, so we failed to adjust it for inflammation, as well as oxidative stress markers. Fourth, we didn’t collect indicators that quantified the nutritional status of patients, such as body mass index (BMI); nonetheless, we did our best to understand patients' lifestyles by SGA and excluded patients with severely abnormal nutritional status before inclusion. Fifth, due to the limitation of sample size, confounders couldn't be adequately adjusted; many other confounding factors, such as daily exercise, smoking, drinking, medications, and family history weren’t adjusted for in our multivariate regression models. Finally, comorbidities of paroxysmal AF, including hypertension, coronary heart disease, and diabetes mellitus, may interfere with the present results, but the limited sample size makes them impossible to exclude. However, we analyzed the difference in ALB levels among the comorbidities of paroxysmal AF and adjusted for them in our logistic models. It would be interesting to investigate the association between ALB levels and lone AF in the future. Ideally, the findings of this study would be validated in a larger cohort.

## Conclusion

In conclusion, our study indicates that low ALB levels in male patients are independently associated with paroxysmal AF. ALB levels are positively associated with TG, TC, HDL-C, and LDL-C. These findings imply that hypoproteinemia and low blood lipid profiles may act synergistically to involve in the pathological development of paroxysmal AF. Moreover, it would be essential to perform a prospective cohort design to investigate the causal relationships between these conditions.

## Data Availability

The datasets used and/or analyzed during the current study are available from the corresponding author on reasonable request.

## Abbreviations

ALB: Albumin
Paroxysmal AF: Paroxysmal atrial fibrillation
TG: Triglyceride
TC: Total cholesterol
LDL-C: Low-density lipoprotein cholesterol
HDL-C: High-density lipoprotein cholesterol
CHD: Coronary heart disease
APOA1: Apolipoprotein A1
APOB: Apolipoprotein B
Lp (a): Lipoprotein (a)
SUA: Serum uric acid
ALT: Alanine aminotransferase
AST: Aspartate aminotransferase
SCr: Serum creatinine
ORs: Odds ratios
CI: Confidence interval
CRP: C-reactive protein
IL-6: Interleukin-6
BMI: Body mass index
